# Using Transformer-Based Topic Modeling to Examine Discussions of Delta-8 Tetrahydrocannabinol: Content Analysis

**DOI:** 10.2196/49469

**Published:** 2023-12-21

**Authors:** Brandi Patrice Smith, Brooke Hoots, Lara DePadilla, Douglas R Roehler, Kristin M Holland, Daniel A Bowen, Steven A Sumner

**Affiliations:** 1 Office of Strategy and Innovation National Center for Injury Prevention and Control Centers for Disease Control and Prevention Atlanta, GA United States; 2 Division of Overdose Prevention National Center for Injury Prevention and Control Centers for Disease Control and Prevention Atlanta, GA United States; 3 US Public Health Service Commissioned Corps Bethesda, MD United States; 4 Division of Violence Prevention National Center for Injury Prevention and Control Centers for Disease Control and Prevention Atlanta, GA United States

**Keywords:** social media, natural language processing, public health surveillance, machine learning, topic modeling, delta-8 tetrahydrocannabinol, cannabis, marijuana

## Abstract

**Background:**

Delta-8 tetrahydrocannabinol (THC) is a psychoactive cannabinoid found in small amounts naturally in the cannabis plant; it can also be synthetically produced in larger quantities from hemp-derived cannabidiol. Most states permit the sale of hemp and hemp-derived cannabidiol products; thus, hemp-derived delta-8 THC products have become widely available in many state hemp marketplaces, even where delta-9 THC, the most prominently occurring THC isomer in cannabis, is not currently legal. Health concerns related to the processing of delta-8 THC products and their psychoactive effects remain understudied.

**Objective:**

The goal of this study is to implement a novel topic modeling approach based on transformers, a state-of-the-art natural language processing architecture, to identify and describe emerging trends and topics of discussion about delta-8 THC from social media discourse, including potential symptoms and adverse health outcomes experienced by people using delta-8 THC products.

**Methods:**

Posts from January 2008 to December 2021 discussing delta-8 THC were isolated from cannabis-related drug forums on Reddit (Reddit Inc), a social media platform that hosts the largest web-based drug forums worldwide. Unsupervised topic modeling with state-of-the-art transformer-based models was used to cluster posts into topics and assign labels describing the kinds of issues being discussed with respect to delta-8 THC. Results were then validated by human subject matter experts.

**Results:**

There were 41,191 delta-8 THC posts identified and 81 topics isolated, the most prevalent being (1) discussion of specific brands or products, (2) comparison of delta-8 THC to other hemp-derived cannabinoids, and (3) safety warnings. About 5% (n=1220) of posts from the resulting topics included content discussing health-related symptoms such as anxiety, sleep disturbance, and breathing problems. Until 2020, Reddit posts contained fewer than 10 mentions of delta-8-THC for every 100,000 cannabis posts annually. However, in 2020, these rates increased by 13 times the 2019 rate (to 99.2 mentions per 100,000 cannabis posts) and continued to increase into 2021 (349.5 mentions per 100,000 cannabis posts).

**Conclusions:**

Our study provides insights into emerging public health concerns around delta-8 THC, a novel substance about which little is known. Furthermore, we demonstrate the use of transformer-based unsupervised learning approaches to derive intelligible topics from highly unstructured discussions of delta-8 THC, which may help improve the timeliness of identification of emerging health concerns related to new substances.

## Introduction

### Emergence of Novel Cannabinoids

Over the last 10 years, the use of cannabis-based products in the United States has increased, with possible reasons including increased legalization, expanding types of products, and wider access to cannabis [1,2]. The passage of the Agriculture Improvement Act of 2018 (Farm Bill) effectively legalized hemp; consequently, novel cannabinoids derived from hemp such as delta-8 tetrahydrocannabinol (THC) emerged in the marketplace [3]. The health effects of novel cannabinoids are not well or widely known. Delta-8 THC is a psychoactive cannabinoid found in small amounts naturally in the cannabis plant and can also be manufactured in larger quantities from hemp-derived cannabidiol (CBD). Most states permit the sale of hemp and hemp-derived CBD products, which typically are not psychoactive in nature [4]. Because delta-8 THC products are derived from hemp, they have become widely available in some state hemp marketplaces. Increasing concerns about the potential unanticipated psychoactive effects (in part given by its availability in marketplaces where cannabis is illegal) and health risks from delta-8 THC warrant the need for more research around its use, trends in popularity, and the types of health effects individuals may experience [5].

### Delta-8 THC and Health Concerns

Emerging evidence from public health agencies further supports the need to study hemp-derived cannabinoids such as delta-8 THC and determine their adverse health outcomes and psychoactive effects that consumers may or may not anticipate [3]. The Centers for Disease Control and Prevention (CDC) and the Food and Drug Administration (FDA) have issued public notices warning of the potentially harmful effects of delta-8 THC. On September 14, 2021, the CDC published a health advisory through the Health Alert Network reporting an increase in adverse health events (eg, lethargy, difficulty breathing, and decreased blood pressure) associated with delta-8 THC [6]. The FDA reported 104 adverse events between December 1, 2020, and February 28, 2022, including hallucinations, dizziness, confusion, and loss of consciousness [7]. Both notices included data from national poison control centers, which reported 2362 delta-8 THC exposure cases between January 1, 2021, and February 28, 2022, including pediatric inpatient and outpatient cases. Furthermore, delta-8 THC products are unregulated and contain byproducts that are not well-characterized; thus, a better understanding of trends in usage and potential adverse health effects could help to avoid public health problems such as the recent e-cigarette, or vaping, product use associated lung injury (EVALI) outbreak associated with an unregulated chemical additive [8,9].

### Social Media and Health Surveillance

Data on cannabis-based product use trends and health outcomes have traditionally come from self-report surveys or interactions with health care providers and clinicians [10,11]. The study of social media data has emerged as a complement to augment traditional public health surveillance [12]. Social media data can help provide real-time information and identify discussions on rare or emerging topics [13]. Social media also allows researchers to see public health concerns from the public’s perspective regarding potential symptomatic and health-related effects that may not be reported in routine medical visits or other traditional health surveillance systems.

This study aims to leverage social media data from Reddit (Reddit Inc) to identify emerging trends and topics discussed about delta-8 THC, including health concerns, adverse symptoms from delta-8 THC use, and other emerging THC products or isomers. We test and use state-of-the-art topic models based on transformer-based neural network architectures used in natural language processing (NLP) [14]. BERTopic, which uses the Bidirectional Encoder Representations From Transformers (BERT) framework, specifically uses transformers, a novel type of neural network model, to detect the coherent context in textual data, such as social media, and cluster-based techniques to determine topics [15]. The analytical approach we use helps inform our current understanding of delta-8 THC and provides a model for future public health efforts to study emerging substances.

## Methods

### Data Source

Reddit is a major social networking site comprised of multiple topical web-based forums and has referred to itself as “the front page of the Internet [16].” Available estimates indicate that Reddit maintains over 50 million daily active users who post content such as images, links, and questions on various topics, including cannabis and substance use [16]. “Subreddits” or topic-specific communities house the designated topics [17].

Pushshift.io provides an application programming interface (API) that allows users to easily query and access Reddit’s historical data [18]. In this study, we identified 115 subreddits related to cannabis, drawn from prior research on drug subreddits and additional qualitative searching of links on the Reddit platform [13]. We obtained the questions and comments (hereafter referred to as posts) from each subreddit starting in January 2008, the earliest date when Reddit enabled user-generated subreddit, to December 2021, using the Pushshift API. Critical data collected included the raw unstructured text from the post and the date of the post.

### Determining Mentions of Delta-8 THC

To first isolate posts about delta-8 THC out of the larger sample of all cannabis and drug-related posts, we identified spelling variants and abbreviations used in discussing delta-8 THC. A Word2Vec model was trained and implemented in PySpark to identify spelling variants [19-21]. Minimal preprocessing of the raw text was performed, as is typical when further downstream use in neural network models is used, with the exception of lowercasing text and removing URL links. This allows neural network models to use all available information, including nonstandard abbreviations or symbols. Word2Vec is a neural network-based machine learning model that converts words to numerical vector representations [20]. We used the resulting word vectors to identify the terms with the highest cosine similarity to delta-8 THC. The authors reviewed these terms to identify the relevant linguistic variations of delta-8 THC (ie, d-8, d8, delta-8-thc, delta-8, d8thc, delta8, d8thc, 8thc, delta8thc, and delta 8). We used this list of terms to filter our data set for posts explicitly mentioning delta-8 THC or synonymous word variations.

### Topic Modeling

Recent advances in unsupervised topic modeling have focused on leveraging models based on transformer architectures and large amounts of pretrained language data, such as the well-known BERT language model released by Google [14]. However, most applications of BERT and transformer models have centered on supervised learning tasks—explicitly teaching the model what categories to classify into by tuning the model using human-labeled data. In this work, we use unsupervised learning capabilities of such models to identify topics discussed in the posts without a priori knowledge of the existing themes. The main benefit of using transformer-based models is that they more efficiently identify contextual relationships between words in large data sets than previous NLP models [22].

For the specific implementation of this unsupervised topic modeling, we use the BERTopic framework, which is available as a Python-based library by the same name [15]. BERTopic uses a probabilistic generative topic model, which uses deep learning models to determine the topics in a collection of documents. BERTopic first represents each post as an embedding, leveraging a large pretrained language model. As a next step, the dimensionality of the embeddings is reduced using uniform manifold approximation and projection, an algorithm that outperforms traditional techniques such as principal component analysis in the setting of high-dimensional data [23]. Finally, the reduced embeddings are clustered via Hierarchical Density-Based Spatial Clustering of Applications with Noise (HDBSCAN) and assigned to topics [15]. The parameters selected in our implementation of BERTopic were as follows: sentence transformer model “all-MiniLM-L6-v2” for the embedding model and min_cluster_size=73, metric=euclidean, cluster_selection_method=eom, and min_samples=5 for HDBSCAN.

As the accuracy of any topic modeling approach depends on selecting the ideal number of clusters or topics needed to describe the data optimally, we determined the ideal number of topics through a grid search of possible minimum cluster sizes. We evaluated each iteration’s UMass coherence score, a widely used metric for this purpose which can be calculated via the Python-based *Gensim* library [24]. Manual inspection of topics or clusters verified the accuracy of the assignment of posts; BERTopic also identifies posts that cannot be reliably assigned to any cluster. This is both a strength and a limitation of the algorithmic approach, as elaborated further in the study. The ability of BERTopic to prevent the inclusion of poorly fitting posts into a given topic helps preserve topic clarity and interpretation of results. However, this also means that some information is unused. Nonetheless, algorithmic approaches are necessary when the volume of information is too large to be reviewed by human annotators. Furthermore, the brevity and informality of social media text can also make it impossible for even human reviewers to accurately assign topics; thus, maintaining a topic categorization scheme that permits a category for content that is unable to be classified is necessary. Finally, we randomly sampled 50 posts from each topic generated and labeled each topic by qualitatively reviewing the posts. The posts were reviewed by 3 reviewers to ensure agreement and accuracy of the assigned topic label. Last, we collapsed topics into significant themes for the presentation of results.

### Ethical Considerations

This study used publicly available data from the Pushshift API, available to all researchers and the public. The study was reviewed by the CDC and deemed exempt from CDC institutional review board review (#6871). Nonetheless, there are important ethical considerations for internet-based research. Example posts that are presented are slightly edited to preserve anonymity. Further, manual qualitative inspection and review of the data found no identifiable information and content included in this study therefore includes no such identifying information.

## Results

**Figure 1:**
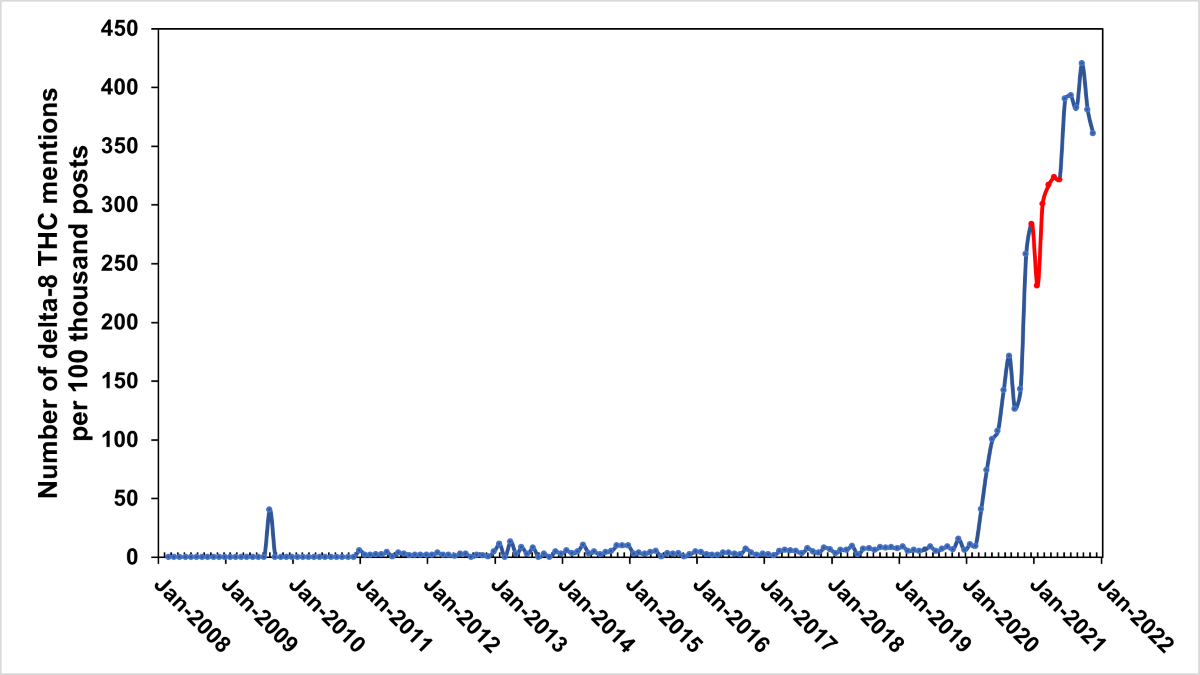
Rate of delta-8 THC mentions in cannabis-related Reddit posts from 2008-2021. The red line highlights the rate of delta-8 THC mentions from January 2021 to July 31, 2021, when self-reported cases of delta-8 THC exposure were recorded with a new product code and reported to the Centers for Disease Control and Prevention health advisory via the National Poison Data System. THC: tetrahydrocannabinol.

In total, there were 44,719,379 posts retrieved from the 115 cannabis-related subreddits from January 2008 to December 2021. Of these, less than 1% (n=41,191) were discussions of delta-8 THC. Figure 1 [6] illustrates the rate (number of mentions per 100,000 posts) of delta-8 THC mentions in cannabis-related posts from 2008 to 2021. Until 2020, Reddit posts contained fewer than 10 mentions of delta-8-THC for every 100,000 cannabis posts annually. However, in 2020 these rates increased by 13 times the 2019 rate to 99.2 posts per 100,000 cannabis posts and continued to increase into 2021 (349.5 mentions per 100,000 posts).

Topic modeling on the 41,191 delta-8 THC-related Reddit posts yielded 81 discrete topics, including a single category that the algorithm returns for posts that the topic modeling could not classify. This topic contained 17,843 (43.3%) posts. Although, this is a large percentage of the total posts, manual qualitative inspection of these posts confirmed the difficulty in classifying this content as discussed in the *Methods* section above. For example, some of these posts were brief phrases without a clear topical focus, such as “upvote d8” or “looks like delta-8,” and we omitted the topic encompassing these posts from further analysis. Additionally, we classified 682 posts into a noninformative category that contained only a subreddit title or the word “delta 8” without any additional information; these were also omitted from further analysis, yielding 22,666 relevant posts.

Of the resulting 22,666 posts, the most common topic, comprising 27.6% (n=6260) of posts, centered on product modalities, such as cartridges, edibles, vapes, tinctures, oils, waxes, gummies, and pens (Table 1). The second most common topic (n=4495, 19.8%) compared delta-8 THC to other cannabinoids, including delta-9 THC, CBD, cannabigerol, THC-O acetate, and hexahydrocannabinol in terms of toxicity, costs, symptoms, and product brands. The third most common topic comprised 13.8% (n=3128) of posts. It included warning posts made by bots, a product feature within Reddit that some subreddits or moderators may use to send automated messages to users. For example, posts on this topic warned Reddit users about the potentially harmful illicit drug market for delta-8 THC products or potential misinformation about lab testing of delta-8 THC brands or products. The legality topic comprised 11.2% (n=2548) of posts. It included discussions about delta-8 THC usage in states where cannabis use is illegal or the implementation of the Farm Bill and its possible effects on delta-8 THC availability and usage. Another 6.1% (n=1390) of posts fell under the topic of general interest in delta-8 THC and the discussions included experience with delta-8 THC products among people who use them and questions from Reddit users who were curious about delta-8 THC more generally. The topic of health made up 5.4% (n=1220) of posts and included discussions about the perceived therapeutic effects of delta-8 THC (eg, reduction in anxiety or sleep issues) and the negative health effects (eg, lung and breathing issues). Each of the following topics comprised less than 5% of posts: lab testing for safety, psychoactive and other effects, chemical nature or properties, product mistrust, purchasing, and drug testing.

Table 2 displays example posts from each of the 12 topic areas to further elucidate the type of content being discussed.

**Table 1:** Distribution of delta-8 tetrahydrocannabinol Reddit posts per topic.

Topics	Posts (n=22,666), n (%)
Specific product discussion	6260 (27.6)
Comparison to other isomers	4495 (19.8)
Bot warning	3128 (13.8)
Legality	2548 (11.2)
General interest in delta-8 THC^a^	1390 (6.1)
Health	1220 (5.4)
Lab testing for safety	1112 (4.9)
Psychoactive and other effects	857 (3.8)
Chemical nature or properties	511 (2.3)
Product mistrust	473 (2.1)
Purchasing	385 (1.7)
Drug testing for work	287 (1.3)

^a^THC: tetrahydrocannabinol.

**Table 2:** Example posts about delta-8 THC^a^ by topic area resulting from BERTopic modeling.

Topics	Quotes from Reddit posts
Specific product discussion	Post 1: “i have tried d8 edibles, and was not disappointed, the few d8 dabs and carts i have tried were not a good first impression, to say the least.” Post 2: “i did 600mg of thc delta 8 gummies my first time ever trying any type of edibles it wasn't even as strong as one puff of weed.”
Comparison to other isomers	Post 1: “d9 is too much for me, i prefer d8.” Post 2: “for me i've felt like using d8 slightly decreases my d9 tolerance.”
Bot warning	Post 1: “a friendly reminder - a lot of the ‘popular’ d8 brands have tested dirty, and some are only popular thanks to intense astroturfing on social media. be sure to avoid [brands of d8 that have tested dirty or have insufficient safety tests] (because they have been [hurting] and even [hospitalizing] people. instead, stick to [delta-8-thc vendors who have safety tests for metals, pesticides, residual solvents, residual acid reagents, and nano silica bleaches (if the brand uses them)].” Post 2: “this post has been locked due to brigading from scammers on reddit who are selling dirty d8 products. a mod will review it asap, and unlock it if it doesn't break any rules. if you are asking about delta 8 thc, [ysk that the “hemp derived” d8 sold online and in headshops has been independently tested and found to be low purity, hot with illegal levels of d9, and have lots of nasty contaminates, on par with the worst dank vapes.] because of this, it is important to stick to delta 8 products that have been fully tested for safety, including for residual reagents, solvents, pesticides, metals, and microbes.”
Legality	Post 1: “currently under the farm bill, only delta 9 thc is regulated. that means that all other cannabinoids, including d8, d10, thcv, thca, thcp, etc are legal.” Post 2: “the likely reason delta-8 isn’t illegal is because the language is unclear and only singles out delta-9. if it’s not federally legalized soon, look for delta-8 to be regulated/prohibited next.”
General interest in delta-8 THC	Post 1: “haven’t used delta 8. What’s up with it??” Post 2: “just requested a few different varieties of the delta-8. what is the experience like?”
Health	Post 1: “my spouse and i took some delta 8 oil, which was too much. we both had irregular heartbeats. I haven’t tried it with delta 9 though.” Post 2: “i hate d8, it doesn’t work medicinally for me. Which sucks cause i wish it did, but i’ve tried every method from dabbing to carts to eating and they’re all awful. It just affects me poorly, it’s a high without a high. also my lungs were insanely bad from it, real dabs have never done that to me. felt toxic.”
Lab testing for safety	Post 1: “lab tested delta 8. that's rare. none of them are lab tested. so good luck but when u have health problems and someone told you not to do it cause there is no upper hand to delta 8. don't say i didn't warn you. delta 8 won't be around long. once they regulate it all products will be gone just like k2 and other stupid stuff they tried to come out with.” Post 2: “there is nothing toxic in d8. it is lab tested most websites you can purchase it from.”
Psychoactive and other effects	Post 1: “it’s actually pretty good. people say you can’t get high off delta 8 but i am a very heavy smoker and enough hits off this and i’ll be in a pretty good state of mind to go to my job without being stupid.” Post 2: “i hate d8, doesn’t work medicinally for me. sucks cause i wish it did, but i’ve tried every mode from dabbing to carts to consuming orally and they’re all awful. just affects me badly, it’s a high without a high. also my lungs were insanely bad from it, real dabs have never done that to me. felt toxic.”
Chemical nature or properties	Post 1: “sounds like it could be delta 8 sprayed hemp flower, which is increasing in popularity lately. everyone should avoid this stuff if possible. it always has high amounts of chemical solvents that weren't eliminated properly.” Post 2: “delta 8 does occur naturally in the plant, but not in quantities required to isolate it. what companies are doing is taking cbd^b^ crude oil and converting into delta8 thc. with natural chemicals and solvents that is and then distilled down to a pure form.”
Product mistrust	Post 1: “if the d8 came with a certificate of analysis illustrating it was clean, then we wouldnt hate on it .... clean brands are permitted to be posted. i know, i know, the brands you are shilling for come with acertificate of analysis, just not one with sufficient safety testing is the problem.” Post 2: “[username removed] particularly sold dark pink distillate and this advises looking for pink specifically. looks awfully suspicious. if i were a skeptic i’d say this is advertising pretending to be an article edit:...definitely a shill account for [username removed].”
Purchasing	Post 1: “were they selling delta 8 products at the dispensary or was that from an online source? excited to hear your feedback or thoughts regarding the product. Thank you for posting.” Post 2: “depending on where you reside, if you’re trying too much to find it, you can go to a smoke shop to get delta 8 or 10. some people complain that it isn’t as strong but it works when you can’t find anything else.”
Drug testing for work	Post 1: “am I wrong in assuming that d8 and d9 have identical metabolites? drug testing measures for metabolites of thc not thc itself” Post 2:“you’ll piss dirty from d8 fyi.”

^a^THC: tetrahydrocannabinol.

^b^CBD: cannabidiol.

## Discussion

### Principal Findings

This study evaluated delta-8 THC posts in a web-based drug forum. We further identified critical topics of discussion using a state-of-the-art unsupervised machine learning approach based on transformer models. This work constitutes a crucial effort in public health as new, unregulated cannabis-related products are rapidly emerging in the marketplace and gaining wide usage, despite a limited understanding of health effects. Our study additionally identified that posts related to delta-8 THC began rapidly increasing in March 2020, which was well before delta-8 THC–related health concerns were identified through traditional public health data sources, suggesting this method may improve the timeliness of surveillance of novel substances [6]. This study further underscores the relevance and applicability of web-based data to serve as a complimentary, leading indicator of potential emerging health threats.

Examining the topics from our modeling approach informs an understanding of the rapid growth in delta-8 THC interest. First, a variety of product formulations and ingestion methods are being promoted for this substance, from smoking to vaping to edibles to syringe-filled distillates [25]. While historically cannabis has been commonly consumed through smoking (eg, joints, blunts, and bongs), other modalities are on the rise (eg, edibles, tinctures, and oils) [26]. In the current marketplace, delta-8 THC is available in many different forms; this may facilitate uptake, given that people who use delta-8 THC can easily find a product in their preferred consumption modality [27].

Growth in the use of delta-8 THC is also facilitated by the hemp policy in the 2018 Farm Bill [28], which Reddit users discuss in the relatively large topic of legality. This loophole allows the sale of hemp-derived THC isomers and other cannabinoids, including delta-8 THC, to be sold in products across all states [3,28]. Delta-8 THC is known to have psychoactive effects, and this is supported by individuals who report in Reddit posts that they experienced feeling “high,” “baked,” or “stoned” [29]. Thus, delta-8 THC products represent an alternative to illicit consumption of traditional THC products. An emerging understanding of the psychoactive effects of delta-8 THC, including (1) apprehension and anxiety; (2) euphoria; (3) loquaciousness; (4) lowering of inhibitions; (5) hunger and thirst; (6) feeling “high;” (7) uncontrollable bursts of laughter or giggles; and (8) drowsiness, languor, lassitude, and a pleasant feeling of fatigue, indicate that they are similar to psychoactive effects of delta-9 THC [29]. Furthermore, considerable increases in mentions of delta-8 THC occurred during the 2019 COVID-19 pandemic, a time in which consumers discuss the use of delta-8 THC to treat mental health problems brought on by related stress or anxiety:

delta 8 has helped so much...covid hit and i have pretty much been high. it got to the point where i was hitting my pen at work, unable to get through the day unless i was stoned.

Several studies support the idea that stress, anxiety, boredom, and wanting to be high during the COVID-19 pandemic increased a person’s desire to use cannabis and nicotine vaping products [30-32].

Although interest in delta-8 THC is elevated, individuals in web-based forums expressed concern about various uncertainties related to the substance, including contamination and safety of products. Lab testing was a key topic of discussion. A recent study analyzed delta-8 THC products such as vaporizers (vapes) and distillates for unknown impurities and found several substances in these products that were not listed on the certificate of analysis [33]. Indeed, a relatively large proportion of the posts we identified were made by bots deployed by moderators to help encourage careful consideration of products.

Health-related discussions about delta-8 THC exhibited nuance. Some posts about the substances referenced the use of delta-8 THC to treat health concerns, while others discussed the negative health effects of the consumption of delta-8 THC. For example, anxiety was a prevalent health issue discussed and some posts credited delta-8 THC for anxiety relief—which may indicate unmet health care needs—while other posts discussed exacerbation of anxiety from delta-8 THC [34,35].

Finally, it should be noted that one of the topics with the most posts compared delta-8 THC to other cannabinoids and related substances. For example, delta-8 THC products are considered by some people who use it as “less strong” than delta-9 THC; thus, people who use delta-8 THC products reported feeling comparably safer using them. Because people who use delta-8 THC may rely on Reddit to understand the nuances of this product compared to other novel cannabinoids, this finding further underscores the importance of accurate health-related information on social media platforms.

### Strengths and Limitations

This study applies a robust, unsupervised topic model, BERTopic, to delta-8 THC–related Reddit posts to identify themes and topics. Unsupervised models perform cluster-based calculations to determine the spatial relationship of words resulting in clusters or topics. We used BERTopic as our topic model for several reasons. First, BERTopic is a topic modeling approach that combines techniques of early probability generative models such as latent Dirichlet allocation (LDA) with the use of semantic embeddings and includes dimension reduction of those embeddings for optimized performance. Additionally, BERTopic has been shown to outperform classic topic models such as LDA, nonnegative matrix factorization (NMF), and correlated topic models (CTM) when measured by topic coherence [15,36]. However, BERTopic may underperform in topic diversity or the percentage of unique words for all topics compared to CTM [15]. Finally, BERTopic is advantageous because of several options for fine-tuning the model with transformer-based state-of-the-art language models.

There are also several limitations to this study. First, Reddit data and user profiles do not contain geographical proxies at the state or local level. Thus, we could not determine if Reddit users resided in states where nonmedical adult use of cannabis is legal, which could give insight into whether adverse health effects related to the use of delta-8 THC products were mentioned more often in legal versus nonlegal states. Similarly, age data were not accessible for Reddit users; thus, inferences about users’ age and public perception of delta-8 THC products and use were not assessed. Nevertheless, studies reveal that individuals selling psychoactive substances do target teenagers and children via social media [37,38]. Furthermore, data from Reddit are not representative of the general population and therefore may not perfectly represent public interest in delta-8 THC. Finally, although BERTopic is a state-of-the-art topic modeling approach, no machine learning–based system is perfect at fully categorizing unstructured text. A reliance on human validation, at least partially, remains an important component of such work and although we manually examined a sample of posts from each topic, even human review is imperfect. It should also be noted that it is possible that some posts may contain more than 1 topical area of discussion. Identifying multiple topics within posts is not currently supported by the BERTopic algorithm and represents an area for future development of NLP models. However, the algorithm did optimize topic categorization by finding the ideal UMass coherence score, indicating that the topic selected was, to the best of our ability, the most suitable one assigned. Nonetheless, ongoing advances in NLP, such as increasingly sophisticated large language models and improved tuning of such models to relevant tasks and data, represent an important area for future work [39].

### Conclusions

This study shows the use of web-based data for monitoring emergent public health concerns related to delta-8 THC. Even if emerging substances are detected early, a central question is whether the emerging substance carries the risk of harm to human health and what the nature of the health threat may be. These questions cannot be quickly answered from existing public health surveillance systems and thus novel data sources such as social media offer potential promise in gathering early insights. The fraction of social media posts that are health-related can be potentially used to gauge the magnitude of health-related concerns. Furthermore, examining the types of health concerns mentioned can inform further studies in clinical settings to obtain more definitive assessments of these potential health harms, particularly since the physiologic and psychoactive effects of a novel substance may not be fully known during periods of early emergence in the population. Second, even more mundane topical categories, such as the products and brands being discussed, can be useful for public health purposes. For example, during the response to the national outbreak of EVALI, a key question was whether certain products or brands were of greatest concern for lung injury. Finally, the topics identified can inform the development of public health communication and education materials. Our results revealed that users did have concerns about product safety and legitimacy and were exploring options to verify those factors in products they were considering using or purchasing. Thus, a better understanding of the discussion themes can help public health communication specialists be more responsive to the questions that are emerging. In summary, our study of a novel topic modeling approach highlights its potential for use in understanding evolving public health topics to help inform public health officials and respond to events in a timely manner.

## Abbreviations

API: application programming interface
BERT: Bidirectional Encoder Representations From Transformers
CBD: cannabidiol
CDC: Centers for Disease Control and Prevention
CTM: correlated topic models
EVALI: e-cigarette, or vaping, product use associated lung injury
FDA: Food and Drug Administration
HDBSCAN: Hierarchical Density-Based Spatial Clustering of Applications with Noise
LDA: latent Dirichlet allocation
NLP: natural language processing
NMF: nonnegative matrix factorization
THC: tetrahydrocannabinol

## Data Availability

The data analyzed during this study are available at the Pushshift repository [40].
